# Lipid raft disruption inhibits the activation of Transient Receptor Potential Vanilloid 1, but not TRP Melastatin 3 and the voltage-gated L-type calcium channels in sensory neurons

**DOI:** 10.3389/fcell.2024.1452306

**Published:** 2024-11-29

**Authors:** Maja Payrits, Balázs Zoltán Zsidó, Andrea Kinga Nehr-Majoros, Rita Börzsei, Zsuzsanna Helyes, Csaba Hetényi, Éva Szőke

**Affiliations:** ^1^ Department of Pharmacology and Pharmacotherapy, Medical School and Centre for Neuroscience, University of Pécs, Pécs, Hungary; ^2^ National Laboratory for Drug Research and Development, Budapest, Hungary; ^3^ Hungarian Research Network, Chronic Pain Research Group, Pécs, Hungary; ^4^ Pharmacoinformatics Unit, Department of Pharmacology and Pharmacotherapy, Medical School, University of Pécs, Pécs, Hungary

**Keywords:** TRPV1, TRPM3, L-type voltage-gated Ca^2+^ channel, CIM0216, FPL 64176, veratridine, lipid raft disruption

## Abstract

Transient Receptor Potential (TRP) ion channels like Vanilloid 1 (TRPV1) and Melastatin 3 (TRPM3) are nonselective cation channels expressed in primary sensory neurons and peripheral nerve endings, which are located in cholesterol- and sphingolipid-rich membrane lipid raft regions and have important roles in pain processing. Besides TRP ion channels a wide variety of voltage-gated ion channels were also described in the membrane raft regions of neuronal cells. Here we investigated the effects of lipid raft disruption by methyl-beta-cyclodextrin (MCD) and sphingomyelinase (SMase) on TRPV1, TRPM3 and voltage-gated L-type Ca^2+^ channel activation in cultured trigeminal neurons and sensory nerve terminals of the trachea. We also examined the mechanism of action of MCD by *in silico* modeling. Disruption of lipid rafts by MCD or SMase did not alter CIM0216-induced TRPM3 cation channel activation and the voltage-gated L-type Ca^2+^ channel activation by FPL 64126 or veratridine neither on trigeminal sensory neurons nor sensory nerve terminals. We provided the first structural explanation with *in silico* modeling that the activation of TRPV1, TRPM3 and voltage-gated L-type Ca^2+^ channels is affected differently by the cholesterol content surrounding them in the plasma membrane. It is concluded that modifying the hydrophobic interactions between lipid rafts and ion channels might provide a selective novel mechanism for peripheral analgesia.

## 1 Introduction

Pain management particularly in cases of chronic neuropathic pain is still an unresolved problem, the presently available drugs (opioid, non-steroidal anti-inflammatory/analgesic drugs, adjuvant analgesics such as antidepressants and antiepileptics) do not provide adequate relief in most cases and do not affect the neurogenic components of the inflammatory processes. Due to a range of severe side effects (gastrointestinal bleeding, diabetes, ulcer formation, obesity) they cannot be administered for longer time periods (Labianca et al., 2012; Zuccaro et al., 2012). Therefore, there is a great need to find novel targets and molecular mechanisms for effective analgesia especially at the periphery.

The Transient Receptor Potential (TRP) superfamily includes nonselective cation channels that are activated by temperature changes, ligand binding and other modifications to the channel protein (Gees et al., 2012; Vay et al., 2012). TRP channels are sensors for different cellular and environmental signals, they are ligand-gated non-selective cation channels having a central pore region permeable to Ca^2+^, Mg^2+^, Na^+^, K^+^, and other cations. Thermosensitive members of the TRP superfamily such as TRPV1, TRPA1, TRPM3 and TRPM8 are mostly expressed on primary sensory neurons and peripheral nerve terminals, where they are activated by mechanical, chemical, or thermal stimuli (Nilius, 2014). Since these ion channels play crucial roles in pain integration and transmission they were identified as potential targets in pain management (Nilius, 2014). Upon channel activation local release of pro-inflammatory neuropeptides (calcitonin gene-related peptide (CGRP), Substance P) induces vasodilation, plasma protein extravasation and immune cell activation, collectively called neurogenic inflammation (Szolcsányi, 2004; Szallasi et al., 2007; Luo et al., 2013; Laing and Dhaka, 2016).

TRP Vanilloid 1 (TRPV1) is a nocisensor plasma membrane protein expressed in the large population of polymodal-type nociceptors gated by painful chemical agents, noxious heat (>43°C), protons (pH < 6.0) and vanilloid-type agonists like capsaicin and resiniferatoxin can activate TRPV1 (Welch et al., 2000; Raisinghani et al., 2005; Bianchi et al., 2006; Gavva, 2008; Cao et al., 2013). The TRP Melastatin 3 (TRPM3) is activated by noxious heat and chemical compounds as the neurosteroid pregnenolone sulphate (PS) (Vriens et al., 2011a) or the synthetic ligand CIM0216 (Held et al., 2015). CIM0216 [racemate of 2-(3,4-dihydroquinolin-1(2H)-yl)-N-(5-methylisoxazol-3-yl)-2-phenylacetamide] is a selective activator of the TRPM3 ion channel with a potency greatly exceeding that of the canonical TRPM3 agonist PS. TRPM3 also shows significant co-expression with TRPV1 (Kashio and Tominaga, 2022). Despite significant efforts to identify potential drug targets, only a few drug candidates targeting specific TRP channels have progressed to clinical stages of drug development (Szolcsányi and Sándor, 2012; Kaneko and Szallasi, 2013; Nilius and Szallasi, 2014) for autoimmune diseases, tumors, and inherited disorders (Nicholson and Verma, 2004; Sacco, 2013).

Several members of the TRP superfamily, including the termo-TRP channels (TRPV1, TRPA1, TRPM3 and TRPM8) are known to be localized in special microdomains of the plasma membrane, called the lipid rafts (Liu et al., 2006; Szolcsányi, 2008). These are dynamic structures containing significant amount of special lipid constituents, with approximately doubled cholesterol and sphingomyelin levels compared to other plasma membrane areas (Fridriksson et al., 1999; Pike et al., 2002), leading to enhanced orderliness and membrane stiffness (Simons and Ikonen, 1997; Simons and Sampaio, 2011; Sonnino and Prinetti, 2013). Lipid rafts play a crucial role in receptor activation by stabilizing receptor position and facilitating receptor assembly (Mishra and Joshi, 2007; Bobkov and Semenova, 2022).

We, and other research groups have already described that lipid raft disruption and targeting of the hydrophobic lipid-protein (membrane-ion channel) interactions lead to decreased activation of TRPV1, TRPA1 and TRPM8 ion channels (Szoke et al., 2010a; Sághy et al., 2015a; Horváth et al., 2020a; Horváth et al., 2021a; Sántha et al., 2020; Nehr-Majoros et al., 2024). The integrity of the rafts can be disrupted in several ways. The two main components of the lipid rafts are sphingolipids and cholesterol which can be depleted from the rafts. Methyl-beta-cyclodextrin (MCD), known as the gold standard for lipid raft disruption, can form host-guest inclusion complex with cholesterol, thus effectively depleting it from the membrane (Eckert, 2010). Sphingomyelinase (SMase) catalyzes the breakdown of sphingomyelin into phosphocholine and ceramide also abolishing membrane order in the raft regions (Cremesti et al., 2002). Our research group described that both MCD and SMase treatments decreased TRPV1 and TRP Ankyrin 1 (TRPA1) ion channel activation via lipid raft disruption *in vitro* in trigeminal ganglion (TRG) neurons and a transfected Chinese Hamster Ovary (CHO) cell line expressing the ion channels (Szoke et al., 2010b; Sághy et al., 2015b). We have also provided evidence that MCD and SMase pretreatment had *in vivo* analgesic effect via TRPV1, TRPA1 and TRPM8 ion channel inhibition (Horváth et al., 2020b; Horváth et al., 2021b; Horváth et al., 2024a). The effects of lipid raft disruption in PS plus CIM0216-induced TRPM3 activation was investigated *in vitro* in intracellular Ca^2+^ measurements in ion channel-expressing HEK293T cells, with no significant change in activation upon lipid raft disruption. *In vivo* acute nocifensive pain experiment with PS plus CIM0216 administration was performed in mice to reveal the effect of cholesterol depletion on TRPM3-mediated pain. However, pretreatment didn’t cause significant decrease in the duration of the nocifensive behavior in mice (Horváth et al., 2024b). Besides TRP channels a wide variety of voltage-gated ion channels were also described to be located in the membrane raft regions of neuronal cells. Controversal observations have been described about the role of lipid raft in the operational properties of voltage-gated ion channels. MCD treatment had no effect on the macroscopic biophysical properties of Ca_v_1.3 in neurons (Licon et al., 2015). The Ca_v_2.1 currents have been increased on cerebellar neurons after lipid raft disruption (Davies et al., 2006) but MCD treatment diminished the activation kinetics of delayed K_v_3.1 currents on NG108-15 neuronal cell line (Huang et al., 2011). On the contrary, another plasma membrane cholesterol depletory agent, the α-synuclein selectively activated Ca_v_2.2 channels in rat neurons (Ronzitti et al., 2014). L-type calcium channels (LTCCs) have been described to be associated with caveolin-1-rich lipid rafts in the cerebellar granule neurons plasma membrane, and the treatment with MCD decreased the phosphorylation level of the LTCC β₂ subunit and the calcium concentration in neurons (Fortalezas et al., 2018). In our previous experiments SMase treatment did not affect the percentage of responsive neurons to KCl ion (Sághy et al., 2015c). The involvement of various K^+^ and Ca^2+^ channels in the KCl-evoked Ca^2+^-influx might reflect the diverse functional impacts of lipid raft disruption on the gating of these channels. However the overall response remained unaltered (Szoke et al., 2010c; Sághy et al., 2015a).

The aim of the present study was to investigate whether lipid raft disruption by MCD and SMase cause any changes in voltage-gated ion channels in cultured trigeminal neurons. We aimed to compare these results with those on the TRPV1 and TRPM3 channel activation. Furthermore, the effect of MCD on peripheral nerve terminals was investigated by measuring capsaicin- and electric field stimulation-induced Ca^2+^-influx-dependent release of CGRP from them. In this study capsaicin and CIM0216 were used for TRPV1 and TRPM3 activation, respectively. We used voltage-gated L-type Ca^2+^ channel activator compounds to reveal the action of lipid raft disruptor on the activation of these channels, therefore we could compare the effects in the Ca-imaging experiments in sensory neuronal cultures with the effects caused by TRPV1 and TRPM3 activation. The neurotoxic steroidal phyto-alkaloid veratridine acts through binding to and causing persistent activation of voltage-gated Na-channels mainly in heart, nerve, and skeletal muscle cell membranes (Denac et al., 2000; Yang et al., 2022). FPL 64176 (methyl 2,5-dimethyl-4-[2-(phenylmethyl)benzoyl]-1H-pyrrole-3-carboxylate) is a specific L-type voltage-gated Ca^2+^ channel modulator (Baxter et al., 1993; Triggle, 2006). We tried to reveal a mechanism of action of MCD on TRPV1, TRPM3 and L-type Ca^2+^ channel by *in silico* modeling. We aimed to map the binding modes of cholesterol to these ion channels using the Wrapper step of the Wrap’n’Shake method.

## 2 Materials and methods

### 2.1 Primary cultures of trigeminal ganglion neurons

Trigeminal ganglion (TRG) cultures were prepared from newborn - aged 1–3 days - NMRI mice. TRG were removed in cold phosphate-buffered saline (PBS). The TRGs were incubated in PBS containing collagenase Type XI (1 mg/mL) for 20 min at 37°C, and then in PBS with DNase I (1,000 units/mL) for 8 min at 37°C. After rinsing with PBS, mechanical dissociation was carried out. Cells were placed on glass coverslips which were coated with poly-D-lysine. In a medium comprising DMEM-low glucose, 5% newborn calf serum, 5% horse serum, 5% FBS, 0.1% penicillin-streptomycin, and 200 ng/mL nerve growth factor (NGF). Cells were kept at 37°C in a humidified environment with 5% CO_2_ (Szoke et al., 2000).

### 2.2 Fluorescent ratiometric technique of intracellular free calcium concentration measurement

Measurements were performed on 1–2 days old TRG neuron cultures. Cells were incubated with a fluorescent Ca^2+^ indicator dye, fura-2-AM (1 µM) for 30 min at 37°C in a solution containing (in mM): NaCl, 122; HEPES, 25; KCl, 3.3; CaCl_2_, 1.3; MgCl_2_, 0.4; KH_2_PO_4_, 1.2; and glucose, 10; (pH 7.3). Following the staining process, cells underwent a 5-min wash with extracellular solution (ECS) containing (in mM): NaCl, 150; HEPES, 10; KCl, 2.5; CaCl_2_x2H_2_O, 1; MgCl2 x 6H_2_O, 2; and glucose, 10; pH 7.3. The swift alteration of solutions from a triple outlet tube was managed using a rapid step perfusion system (product code VC-77SP, Warner Instrument Corporation, Harvard Apparatus GmbH, Germany). Calcium transients were assessed via microfluorimetry as outlined in previous studies (Szoke et al., 2000).

Fluorescent imaging was conducted utilizing an Olympus LUMPLAN FI/ × 20 0.5 W water immersion objective and a digital camera (CCD, SensiCam PCO, Germany). The fluorescence of a maximum of 10–14 dye-laden cells per plate was observed. A monochromator (Polychrome II., Till Photonics, Germany) emitted 340 and 380 nm light alternately (each for 100–200 ms) for cell illumination, controlled by Axon Imaging Workbench 2.1 (AIW, Axon Instruments, CA) software. The emitted light was recorded at wavelength >510 nm. The mean fluorescence ratio (R) = F340/F380 was continuously monitored (at a rate of 1 Hz) for up to 2 min. R values generated by AIW 2.1 software were subsequently processed using Origin software version 8.0 (Originlab Corp., Northampton, MA). The peak magnitude of the ratiometric response was measured.

The neurons were incubated with MCD (3 or 10 mM) or SMase (10 or 30 mUN) respectively, for 45 min at 37°C in a humidified atmosphere with 5% CO_2_. Capsaicin (330 nM) and CIM0216 (5 µM) were administered for 10 s to activate TRPV1 and TRPM3 channels, respectively. FPL 64176 (10 µM) and veratridine (20 µM) were added for 10 s to activate voltage-gated Ca^2+^ channels.

### 2.3 Measurement of CGRP release from the peripheral terminals of primary sensory neurons in response to TRPV1 activation or electric field stimulation

The procedure has been thoroughly delineated elsewhere (Helyes et al., 1997a; Németh et al., 1998). To summarize, 6–8 month old Wistar rats were exsanguinated under profound anesthesia (sodium thiobarbital 50 mg kg-1 i.p.), subsequently, tracheae were excised, purified of adipose tissue, and adherent connective materials. The trachea serves as an ideal model to investigate the activation of peripheral nerve endings, given the proximity of peptidergic sensory nerve terminals to the surface, and they can be easily stimulated by agonists (Helyes et al., 2001). Tracheae were placed into an organ bath to induce the release of peptides and were perfused (1 mL/min) with pH 7.2 controlled oxygenated Krebs solution for 60 min (equilibration phase) at 37°C, then incubated with MCD (10 μM, 100 μM, 1 mM) or the vehicle for MCD solely. Following stop of the perfusion, the medium was replaced three times for 8 min each to generate pre-stimulated, stimulated, and post-stimulated fractions. Chemical stimulation was conducted during the second 8-min interval using the selective TRPV1 agonist capsaicin (100 nM or 1 µM) or electric field stimulation (40 V, 0.1 ms, 2 Hz, 50 s) was used to evoke CGRP release. CGRP concentrations were quantified from 200 µL samples of organ fluid utilizing radioimmunoassay technique established in our laboratories. CGRP release measured in the stimulated and post-stimulated fractions was summed, and the basal release measured in the pre-stimulated 8-min fraction was subtracted from this total to determine the net peptide release. CGRP release was expressed in fmol/mL. Three independent experiments were conducted in each group, with 12 tracheae investigated per experiment (2 tracheae in each organ bath chamber) yielding n = 3 × 6 data points per group (Helyes et al., 1997b; Németh et al., 1998).

### 2.4 Statistical analysis

In all cases, results were obtained from a minimum of three independent experiments. Data were evaluated using GraphPad Prism 8.0.1 (GraphPad, La Jolla, CA, United States). Statistical analysis was performed using normality testing, followed by one-way ANOVA to compare control and treated groups, with Dunnett’s *post hoc* test.

### 2.5 Modeling studies

#### 2.5.1 Ligand preparation

Cholesterol was built in Maestro (Schrödinger Release, 2024: Maestro, Schrödinger, LLC, New York, NY, 2024) including the addition of hydrogens and a built-in energy minimization with the software. Gasteiger-Marsili partial charges (Gasteiger and Marsili, 1980) were added in AutoDockTools (Morris et al., 2009).

#### 2.5.2 Target preparation

Atomic coordinates of the human TRPV1 and TRPM3 ion channels and human L-type voltage-gated calcium channel (Ca_v_1.3) in ligand free (apo) form were obtained from the Protein Data Bank (PDB (Berman et al., 2000)) with PDB codes 7l2h (Zhang et al., 2021), 8ed7 (Zhao and MacKinnon, 2023) and 7uhg, respectively. The intracellular parts of the proteins were removed. Missing amino acid residues in the transmembrane region were built with SWISS-MODEL (Waterhouse et al., 2018). Hydrogens and Gasteiger-Marsili partial charges were added to both proteins in AutoDockTools.

#### 2.5.3 Wrap’n’Shake protocol

The Wrapper part of the Wrap’n’Shake (Bálint et al., 2017) method was used to build a monomolecular layer of cholesterol molecules on the target surfaces. The grid maps of the targets were prepared with AutoDockTools software. The grid boxes were placed to cover the entire transmembrane regions of the proteins. The extracellular and intracellular parts were not included in the docking calculations. The size of the grid boxes were 126 × 126 × 36 with a grid spacing of 0.825, and the boxes were centered on the coordinates: 131.5, 132.6, 110.3 (TRPV1), 213.7, 213.3, 170.9 (TRPM3) and 152.5, 156.5, 149.5 (Ca_v_1.3), respectively. The number of docking cycles were set to 30 according to the original publication. The other settings were set to default, described in details in (Bálint et al., 2017). The exit criteria of the protocols are either structural or energetic. The structural exit criterion was reached in the case of the TRPM3 ion channel in the 16th cycle, as no free surface area was detected on the ion channel at the end of the 16th cycle. The Ca_v_1.3 calculation was terminated after 30 cycles, as the cholesterol copies were placed further than 3.5 Å from the target. The energetic exit criterion (E_inter_ > +1 kcal/mol) was reached in the 30th cycle in the case of TRPV1. The docked binding modes were clustered into ranks as described in (Bálint et al., 2017), resulting in 194 (TRPM3), 422 (TRPV1), and 471 (Ca_v_1.3) cholesterol copies, respectively. The cholesterol copies with a distance larger than 3.5 Å from the target were deleted (trimmed) in both cases, and finally 154 (TRPM3), 204 (TRPV1), and 237 (Ca_v_1.3) cholesterol copies remained in the monomolecular layers, respectively. The calculated interaction energy (E_inter_) values of all docked cholesterol copies were collected from the docking output files and evaluated statistically.

#### 2.5.4 Descriptive statistical evaluation

Histograms were produced from the E_inter_ values of the ligand copies for all ion channels. The histogram bin width was set to 0.5 kcal/mol, and a total of 23 bins were set to cover the E_inter_ range between −9.5 and +2.0 kcal/mol.

### 2.6 Animals and ethics

The animals were kept in standard plastic cages at 24°C–25°C, under a 12–12 h light-dark cycle and provided with standard rodent chow and water *ad libitum*. This study uses strains obtained from the Laboratory Animal House of the Department of Pharmacology and Pharmacotherapy, University of Pécs. The Animal Welfare Commettee of University of Pécs did not require the study to be reviewed or approved by an ethics committee according to Law No. XXVIII of 1998 on “Animal protection,” the Decree No. 40 of 2013 (II. 14.) Korm. “On animal testing” issued by the Hungarian Government, and the Directive 2010/63/EU of the European Parliament and of the Council on the protection of animals used for scientific purposes, because the authors used tissues and organs for the *in vitro* experiments.

## 3 Results

### 3.1 MCD inhibited the TRPV1, but not the TRPM3 ion channel activation-mediated Ca^2+^-influx in cultured TRG neurons

**FIGURE 1:**
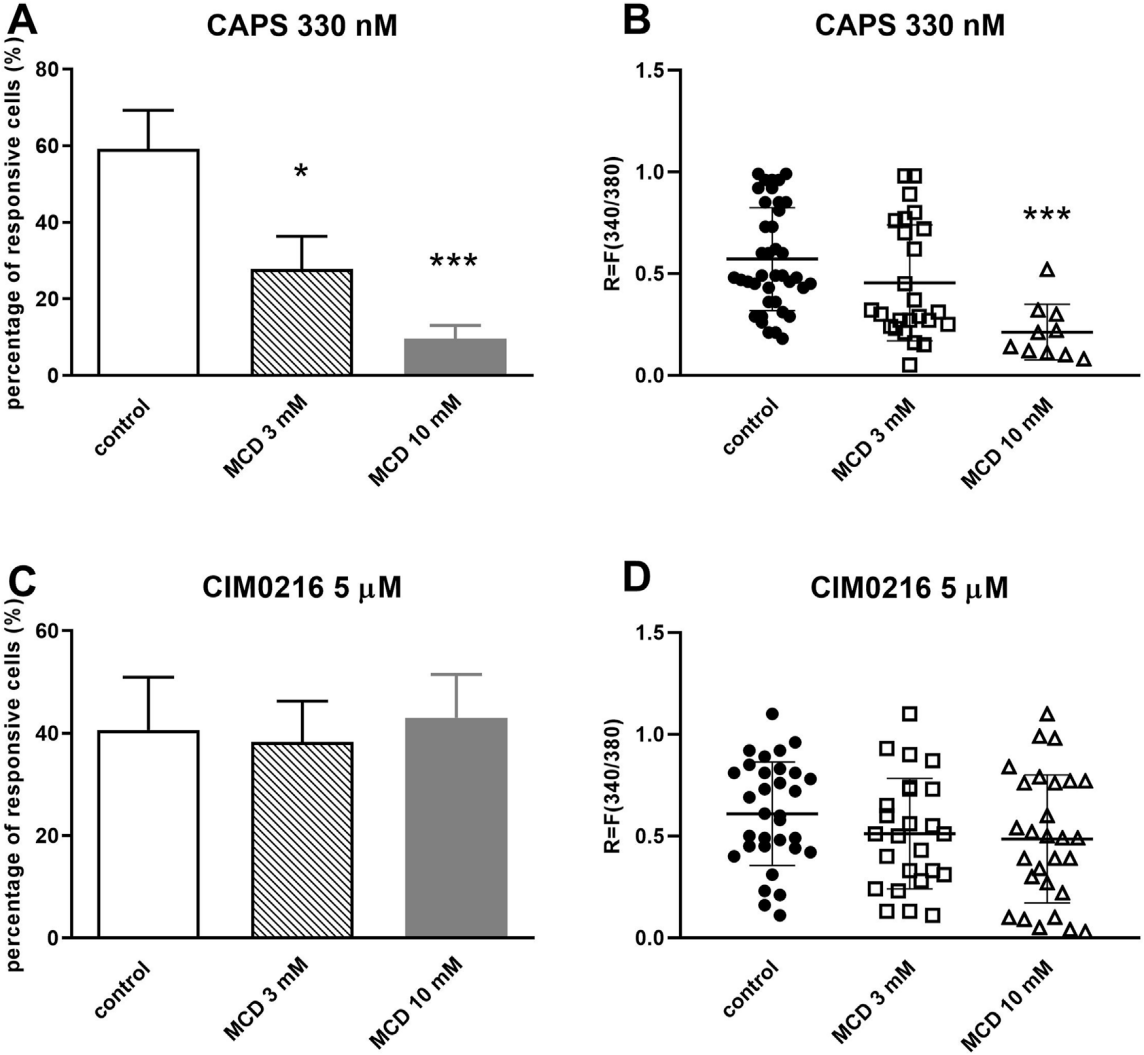
MCD inhibited the TRPV1, but not the TRPM3 ion channel activation-mediated Ca^2+^-influx in cultured TRG neurons **(A)**. Effect of 3 and 10 mM MCD on 330 nM CAPS-evoked TRPV1 ion channel activation in cultured TRG neurons, Ca^2+^-responses are presented in % of total number of examined neurons, *p < 0.05 and ***p < 0.001 (vs. CAPS control), n = 66–103 cells per group. **(B)**. Change in the fluorescence ratio (R = F340/F380) to CAPS is presented after 3 and 10 mM MCD treatment. Dot plot represents mean ± SEM. ***p < 0.001 (control vs. MCD treated). **(C)**. Effect of 3 and 10 mM MCD on 5 µM CIM0216-evoked TRPM3 ion channel activation, (vs. CIM0216 control), n = 64–79 cells per group. No significant decrease in Ca^2+^-influx is detected. **(D)**. Change in the fluorescence ratio to CIM0216 is presented after 3 and 10 mM MCD treatment. Dot plot represents mean ± SEM, control vs. MCD treated. No significant decrease in fluorescence ratio is detected. One-Way ANOVA, Dunnett’s test for multiple comparisons.

Administration of 330 nM TRPV1 agonist capsaicin (CAPS) induced transient Ca^2+^-accumulation in the cytosol of TRG neurons as detected by the magnitude of the fluorescence response. The percentage of responsive neurons to CAPS was determined in control conditions and in the present of 3 or 10 mM MCD. On control plates Ca^2+^-influx was detected in 59.18% ± 10.12% (39 out of 66) of the neurons. After 3 mM MCD treatment this value was decreased significantly to 27.85% ± 8.54% (25 out of 90), and higher concentrations of MCD (10 mM) caused stronger decrease in the proportion of cells responding to CAPS, this value was 9.67% ± 3.47% (10 out of 103) (Figure 1A). The intensity of CAPS-induced fluorescence response was R = 0.57 ± 0.25 on control plates. Incubation with 3 mM MCD did not alter this response (R = 0.45 ± 0.28), while 10 mM MCD significantly attenuated the intensity of CAPS-evoked response resulted in R = 0.21 ± 0.14 fluorescence ratio (Figure 1B).

In the other experimental design, the percentage of neurons responsive to 5 µM CIM0216 (TRPM3 agonist) was determined in control and 3 mM or 10 mM MCD-treated plates. On control plates Ca^2+^-influx was detected in 40.61% ± 10.31% (32 out of 79) of the neurons. After 3 and 10 mM MCD treatment this value did not change significantly (38.3% ± 7.98%, 25 out of 64) and 43.03% ± 8.45%, 28 out of 65); Figure 1C). Furthermore, there was no change in the CIM0216-induced fluorescence intensity after MCD treatment (Figure 1D).

### 3.2 MCD did not inhibit the voltage-gated calcium channel activation in cultured TRG neurons

**FIGURE 2:**
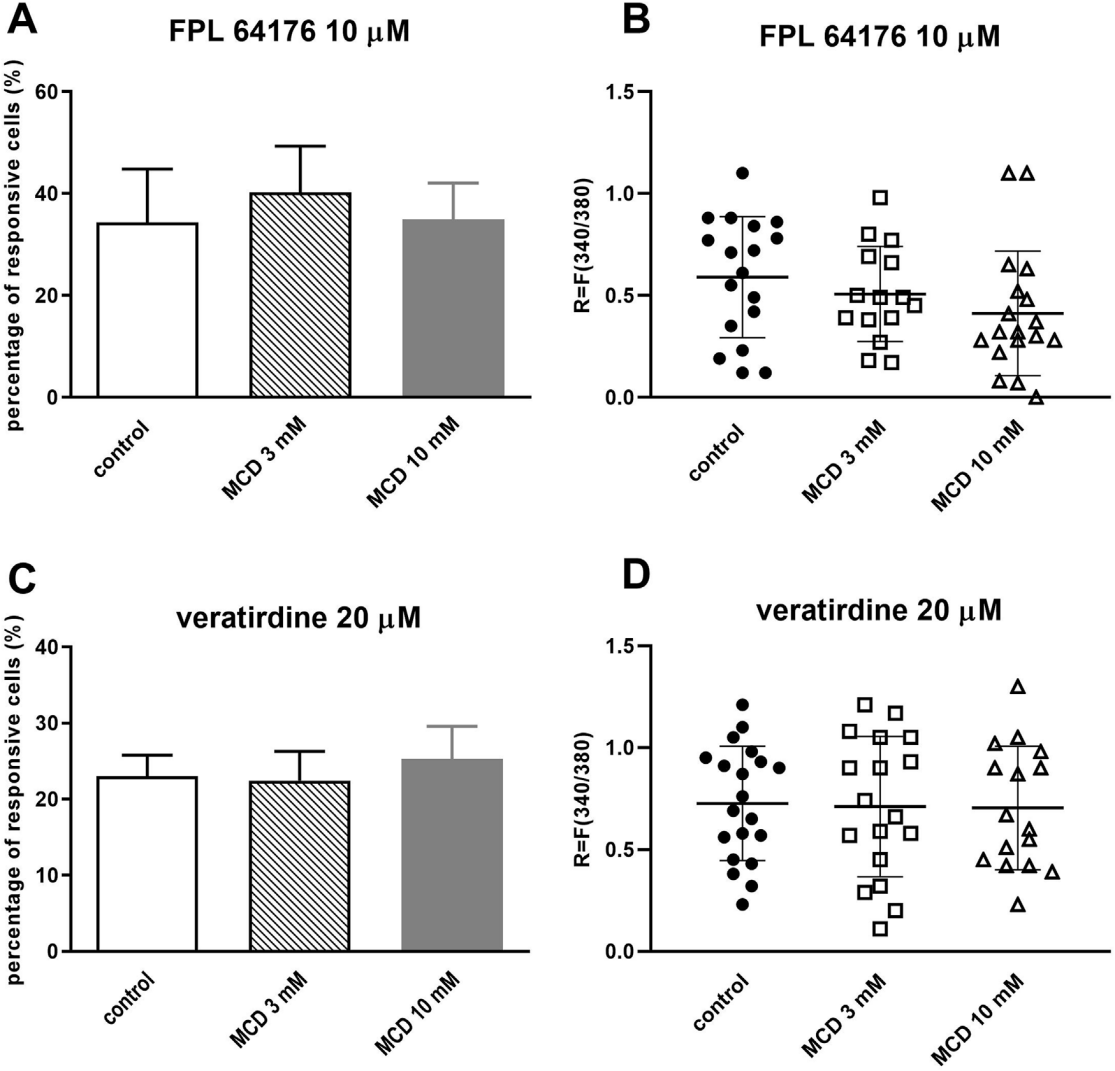
MCD did not inhibit the voltage-gated calcium channel activation in cultured TRG neurons **(A)**. Effect of 3 and 10 mM MCD on 10 µM FPL 64176-evoked voltage-gated Ca^2+^ channel activation in cultured TRG neurons, Ca^2+^-responses are presented in % of total number of examined neurons, (vs. FPL 64176 control), n = 37–52 cells per group. No significant decrease in Ca^2+^-influx is detected. **(B)**. Change in the fluorescence ratio (R = F340/F380) to FPL 64176 is presented after 3 and 10 mM MCD treatment. Dot plot represents mean ± SEM, control vs. MCD treated. No significant decrease in fluorescence ratio is detected. **(C)**. Effect of 3 and 10 mM MCD on 20 µM veratridine-evoked voltage-gated Ca^2+^ channel activation in cultured TRG neurons, (vs. veratridine, +r control), n = 63–87 cells per group. No significant decrease in Ca^2+^-influx is detected. **(D)**. Change in the fluorescence ratio to veratridine is presented after 3 and 10 mM MCD treatment. Dot plot represents mean ± SEM, control vs. MCD treated. No significant decrease in fluorescence ratio is detected. One-Way ANOVA, Dunnett’s test for multiple comparisons.

After pretreating TRG neurons with MCD 3 and 10 mM, we detected the Ca^2+^-influx. The activation was induced by FPL 64176 (10 µM) or veratridine (20 µM). 10 μM FPL 64176 induced Ca^2+^-influx in 34.38% ± 10.46% (18 out of 52) of TRG neurons in control plates and neither 3 mM MCD (40.22% ± 9.12%, 15 out of 37) nor 10 mM MCD treatment (34.98% ± 7.11%, 18 out of 51) caused a difference in the percentage of responding cells (Figure 2A). There was no detectable change in the FPL 64176-induced fluorescence intensity ratio after MCD incubation (Figure 2B).

MCD treatment did not cause significant decrease in the proportion of cells responding to 20 µM veratridine (control plate: 23% ± 2.79%, 20 out of 87; 3 mM MCD: 22.45% ± 3.82%, 18 out of 80; 10 mM MCD 25.32% ± 4.26%, 16 out of 63; Figure 2C). There was no difference in R values in response to veratridine in control and MCD-incubated neurons (Figure 2D).

### 3.3 SMase inhibited the TRPV1, but not the TRPM3 ion channel activation-mediated Ca^2+^-influx in cultured TRG neurons

**FIGURE 3:**
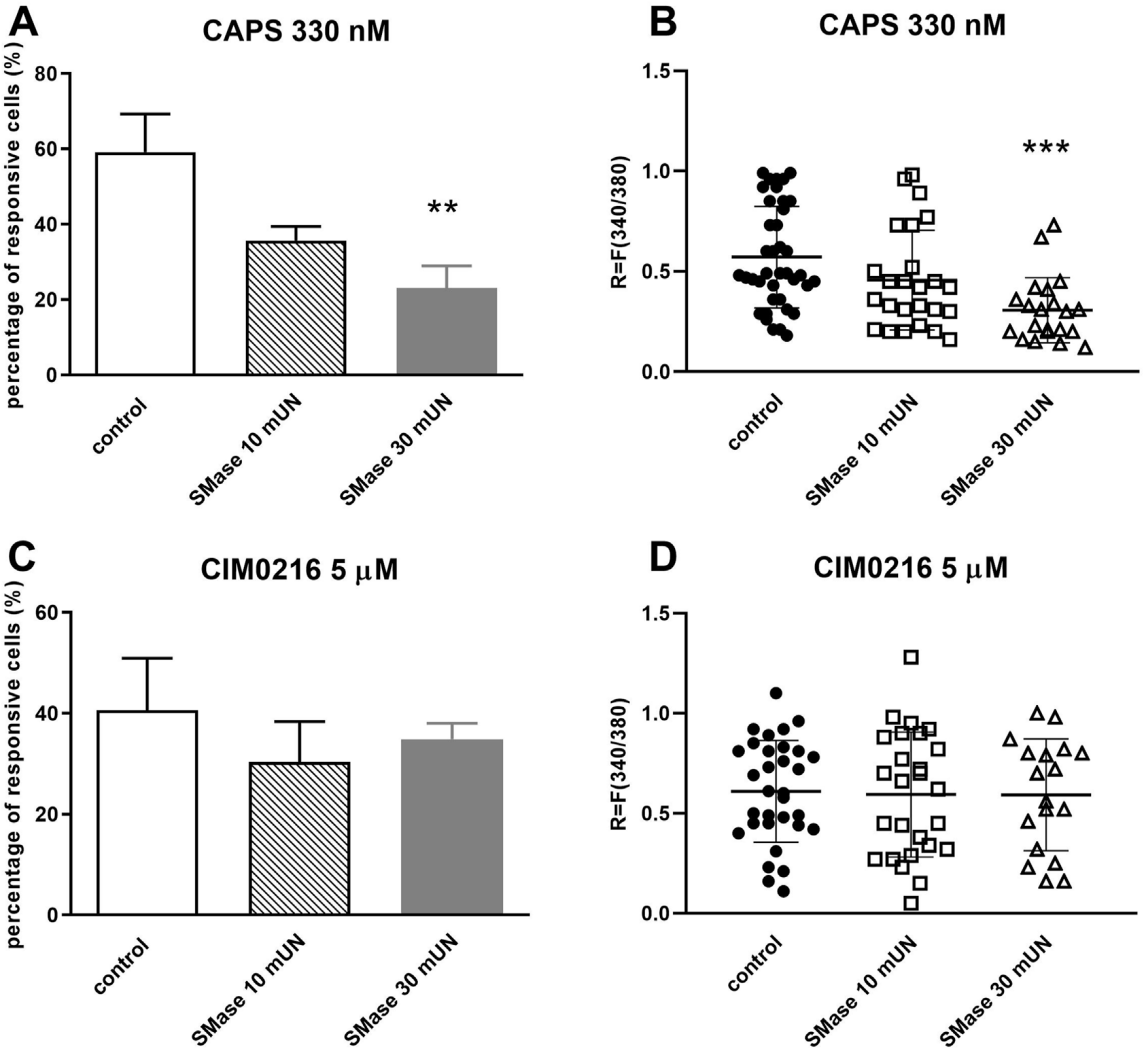
SMase inhibited the TRPV1, but not theTRPM3 ion channel activation-mediated Ca^2+^-influx in cultured TRG neurons **(A)**. Effect of 10 and 30 mUN SMase on 330 nM CAPS-evoked TRPV1 ion channel activation in cultured TRG neurons, Ca^2+^-responses are presented in % of total number of examined neurons, **p < 0.01 (vs. CAPS control), n = 66–91 cells per group. **(B)**. Change in the fluorescence ratio (R = F340/F380) to CAPS is presented after 10 and 30 mUN SMase treatment. Dot plot represents mean ± SEM. ***p < 0.001 (control vs. SMase treated). **(C)**. Effect of 10 and 30 mUN SMase on 5 µM CIM0216-evoked TRPM3 ion channel activation, (vs. CIM0216 control), n = 52–86 cells per group. No significant decrease in Ca^2+^-influx is detected. No significant decrease in Ca^2+^-influx is detected. **(D)**. Change in the fluorescence ratio to CIM0216 is presented after 10 and 30 mUN SMase treatment. Dot plot represents mean ± SEM, control vs. SMase treated. No significant decrease in fluorescence ratio is detected. One-Way ANOVA, Dunnett’s test for multiple comparisons.

Following CAPS activation, the percentage of responsive cells was 59.18% ± 10.12% (39 out of 66), as we already mentioned above, which was reduced by 10 mUN SMase (35.72% ± 3.69%, 25 out of 70), and significantly inhibited by 30 mUN SMase treatment (23.1% ± 5.87%, 21 out of 91) (Figure 3A). The same result was seen in the fluorescence intensity values, 30 mUN SMase caused a significant decrease in the R values (control: R = 0.57 ± 0.25; SMase 10 mUN: R = 0.46 ± 0.25, SMase 30 mUN: R = 0.31 ± 0.16) (Figure 3B). In contrast, SMase had no inhibitory effect on CIM0216-induced Ca^2+^-influx in any applied concentrations (control: 40.61% ± 10.31%, 32 out of 79; SMase 10 mUN: 30.34% ± 8.03%, 26 out of 86; SMase 30 mUN: 34.9% ± 3.11%, 18 out of 52; Figure 3C). The CIM0216-induced fluorescence increment was not inhibited either by SMase (Figure 3D).

### 3.4 SMase did not inhibit the voltage-gated calcium channel activation in cultured TRG neurons

**FIGURE 4:**
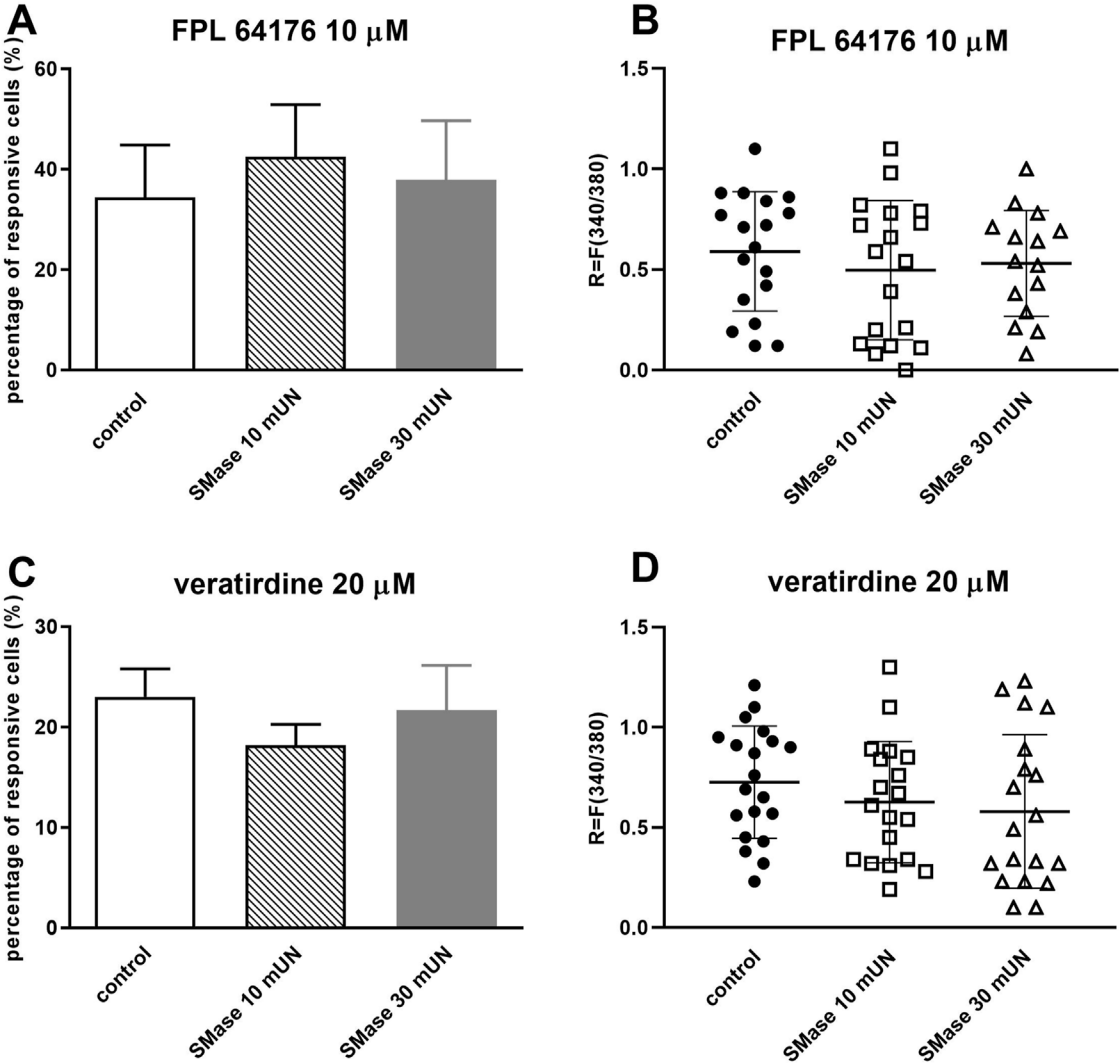
SMase did not inhibit the voltage-gated calcium channel activation in cultured TRG neurons **(A)**. Effect of 10 and 30 mUN SMase on 10 µM FPL 64176-evoked voltage-gated Ca^2+^ channel activation in cultured TRG neurons, Ca^2+^-responses are presented in % of total number of examined neurons, (vs. FPL 64176 control), n = 40–52 cells per group. No significant decrease in Ca^2+^-influx is detected. **(B)**. Change in the fluorescence ratio (R = F340/F380) to FPL 64176 is presented after 10 and 30 mUN SMase treatment. Dot plot represents mean ± SEM, control vs. SMase treated. No significant decrease in fluorescence ratio is detected. **(C)**. Effect of 10 and 30 mUN SMase on 20 µM veratridine-evoked voltage-gated Ca^2+^ channel activation in cultured TRG neurons, (vs. veratridine, +r control), n = 87–105 cells per group. No significant decrease in Ca^2+^-influx is detected. **(D)**. Change in the fluorescence ratio to veratridine is presented after 10 and 30 mUN SMase treatment. Dot plot represents mean ± SEM, control vs. SMase treated. No significant decrease in fluorescence ratio is detected. One-Way ANOVA, Dunnett’s test for multiple comparisons.

SMase incubation with both concentrations did not cause significant decrease in the proportion of cells responding to 10 µM FPL 64176 (control: 34.38% ± 10.46%, 18 out of 52; SMase 10 mUN: 42.48% ± 10.4%, 18 out of 42; SMase 30 mUN: 37.88% ± 11.81%, 15 out of 40, Figure 4A). There was no detectable change in the FPL 64176-induced fluorescence intensity ratio after SMase incubation (Figure 4B).

SMase treatment did not cause significant decrease in the proportion of cells responding to 20 µM veratridine (control plate: 23% ± 2.79%, 20 out of 87; SMase 10 mUN: 18.18% ± 2.08% (19 out of 105) and SMase 30 mUN: 21.68% ± 4.45% (19 out of 88); Figure 4C). There was no difference in ratio values in response to veratridine in control and SMase-incubated neurons (Figure 4D).

### 3.5 MCD inhibited the TRPV1 ion channel activation-, but not the electric field stimulation-mediated CGRP release from peripheral sensory nerve endings

**FIGURE 5:**
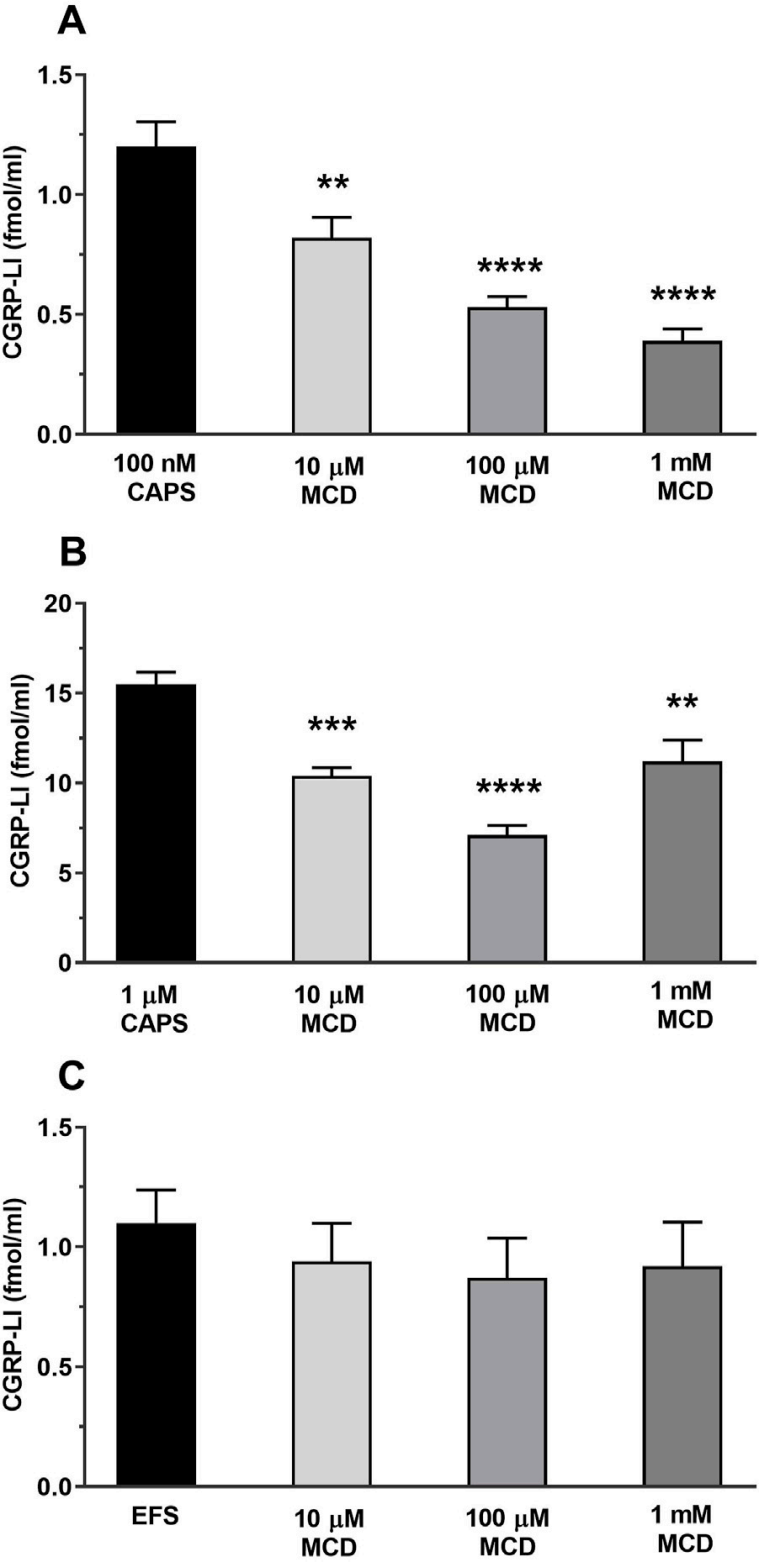
MCD inhibited the TRPV1 ion channel activation-, but not the electric field stimulation-mediated CGRP release from peripheral sensory nerve endings **(A)**. Effect of 10, 100 μM and 1 mM MCD on 100 nM CAPS-evoked CGRP release. **p < 0.01 and ****p < 0.0001 (vs. 100 nM CAPS control), n = 6 preparations per group. **(B)**. Effect of 10, 100 μM and 1 mM MCD on 1 µM CAPS-evoked **p < 0.01, ***p < 0.001 and ****p < 0.0001 (vs. 1 μM CAPS control), n = 6 preparations per group. **(C)**. Effect of 10, 100 μM and 1 mM MCD on EFS-evoked CGRP release. n = 6 preparations per group. No significant decrease in CGRP release is detected, control vs. MCD-treated. One-Way ANOVA, Dunnett’s test for multiple comparisons.

TRPV1 activation by 100 nM CAPS induced 1.2 ± 0.1 fmol/mL CGRP release from peripheral sensory nerve terminals which was significantly and concentration-dependently decreased by MCD. The CGRP release has been reduced after 10 µM MCD treatment to 0.82 ± 0.09 fmol/mL, 100 µM MCD further reduced it: 0.53 ± 0.04 fmol/mL and 1 mM MCD caused 0.39 ± 0.05 fmol/mL CGRP release (Figure 5A). The MCD treatment inhibited the 1 µM CAPS stimulation-evoked CGRP release too at all three applied MCD concentrations from 15.5 ± 0.65 fmol/mL to 10.4 ± 0.45 fmol/mL in 10 µM MCD, and 7.1 ± 0.53 fmol/mL in 100 µM MCD and 11.2 ± 1.18 fmol/mL in 1 mM MCD (Figure 5B).

In the following experimental setup for electric field stimulation 1.1 ± 0.14 fmol/mL CGRP release occurred, which was not significantly altered by MCD incubation. The values were 0.94 ± 0.16, 0.87 ± 0.17 and 0.92 ± 0.18 fmol/mL after using 10, 100 μM and 1 mM MCD, respectively (Figure 5C).

### 3.6 Binding mode of cholesterol to TPRV1 and TRPM3

**FIGURE 6:**
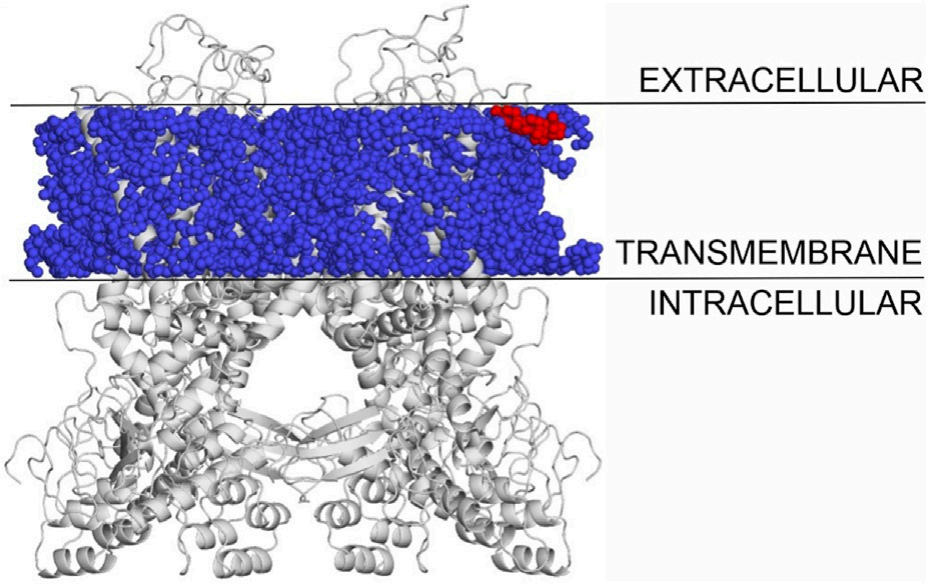
The transmembrane region of TRPV1 covered with cholesterol molecules using the Wrap’n’Shake method. TRPV1 is shown as gray cartoon, cholesterol molecules as blue spheres and the representative cholesterol molecule of the most populated bin is highlighted with red color exposed on the surface on the extracellular side.

**FIGURE 7:**
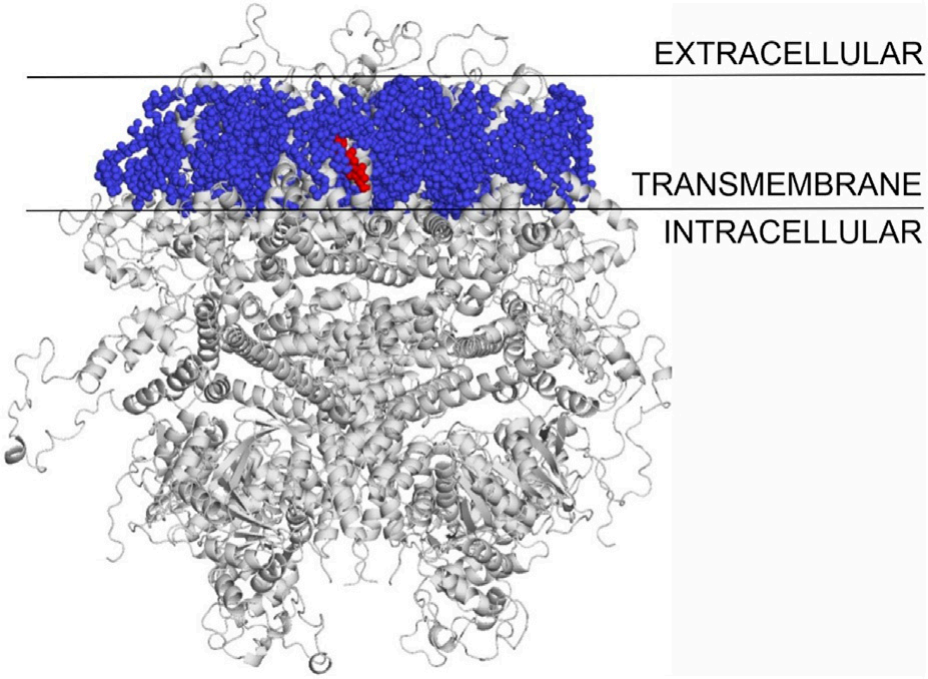
The transmembrane region of TRPM3 covered with cholesterol molecules using the Wrap‘n’Shake method. TRPM3 is shown as gray cartoon, cholesterol molecules as blue spheres and the representative cholesterol molecule of the most populated bin is highlighted with red color buried between the transmembrane helices of the protein.

**FIGURE 8:**
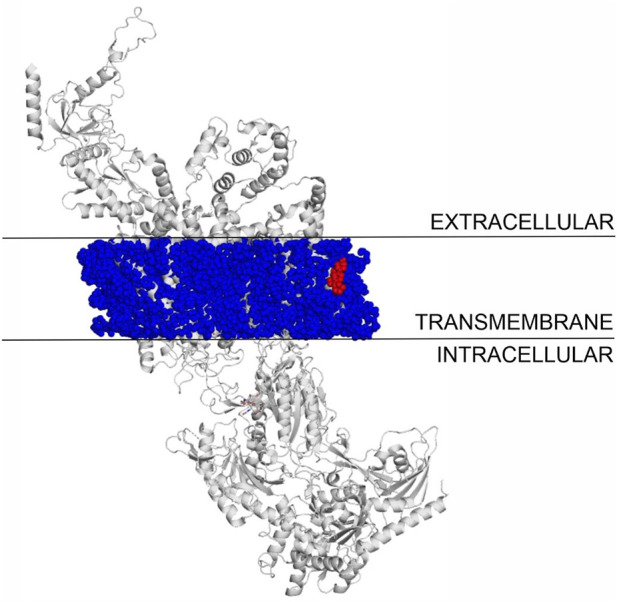
The transmembrane region of Ca_v_ 1.3 covered with cholesterol molecules using the Wrap’n’Shake method. Ca_v_ 1.3 is shown as gray cartoon, cholesterol molecules as blue spheres and the representative cholesterol molecule of the most populated bin is highlighted with red color in the transmembrane region of the protein.

**FIGURE 9:**
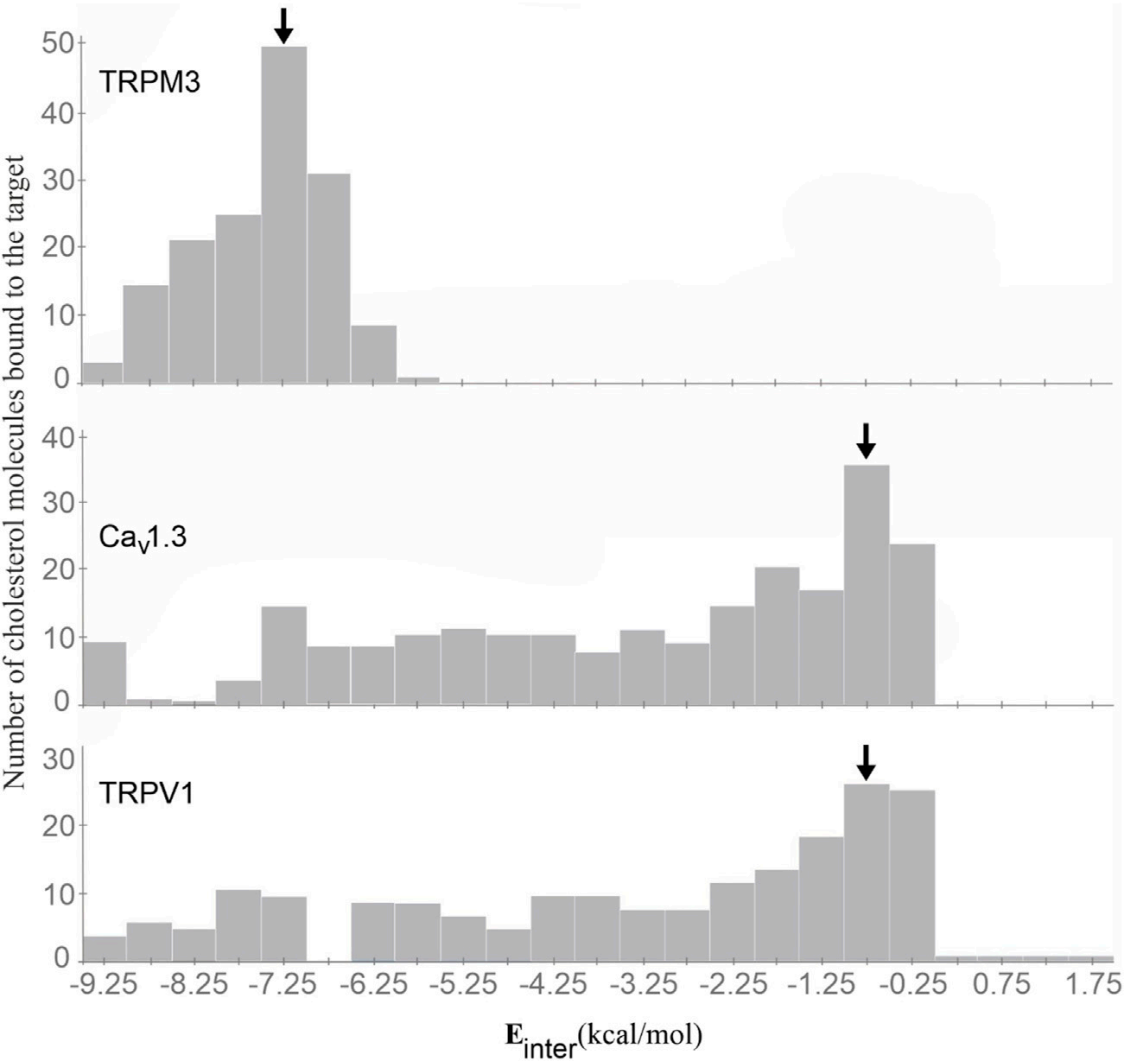
The E_inter_ fingerprints of cholesterol on the TRPM3 (top), Ca_v_ 1.3 (middle), and TPRV1 (bottom) proteins represented as histograms. The most populated bins are highlighted by black arrows. The representative cholesterol molecules were selected from the highlighted bins in all three cases.

The binding modes of cholesterol to TRPV1, TPRM3 and Ca_v_ 1.3 proteins were systematically mapped using the Wrapper step of the Wrap’n’Shake method (Bálint et al., 2017). In all three cases the whole surface of the transmembrane regions of the proteins were covered with ligand copies (Figures 6–8). The corresponding E_inter_ fingerprints of cholesterol were represented as histograms (Figure 9) for all three channels. The comparison of the fingerprints shows (Figure 9) that a radically different E_inter_ pattern was obtained for the proteins. The representative binding modes (Figure 6) of the most populated bins were located between −7.5 and −7.0 kcal/mol for TRPM3 and -1.0 to −0.5 kcal/mol for TRPV1 and Ca_v_ 1.3, with 49, 27, and 35 ligand copies, respectively. Thus, the E_inter_ fingerprints show that there is a considerably larger overall binding affinity (and larger number) of cholesterol molecules stabilizing the structure of TRPM3 if it is compared with TRPV1 and Ca_v_ 1.3. Moreover, in the case of TRPM3 the representative cholesterol binding mode of the above-mentioned most populated bin was buried in the transmembrane helices hidden from the surface (Figure 7). In the case of TRPV1 the representative ligand copy was on the extracellular part exposed on the surface that is easily accessible for the cyclodextrin molecules during the depletion process. In the case of Ca_v_ 1.3 the loosely bound representative cholesterol was also buried in the transmembrane region (similar to TRPM3, Figure 8) of the ion channel. Furthermore, the relatively large extracellular lobe of Ca_v_ 1.3 also hinders the access to the transmembrane region. Thus, the representative cholesterol binding mode of Ca_v_ 1.3 is not as easily accessible as that of TPRV1. However, it is not as strongly bound and buried as that of the TRPM3.

## 4 Discussion

In the present series of experiments we compared the effect of lipid raft disruption on the ligand gated ion channels as TRPV1 and TRPM3 and the voltage-gated L-type Ca^2+^ channels. These are the first data showing that disruption of membrane lipid rafts by depleting cholesterol with MCD or cleaving, +a SMs with SMase inhibits TRPV1 without affecting the CIM0216-induced TRPM3 cation channel activation and the voltage-gated L-type Ca^2+^ channel activation by FPL 64126 or veratridine neither on trigeminal sensory neurons nor sensory nerve terminals. These results are in contrast with our previously described observations regarding the activation of TRPV1, TRPA1, and TRPM8 ion channels following treatment with lipid raft disruptors (Szőke et al., 2010c; Sághy et al., 2015b). We provided evidence for the first time with *in silico* modeling that the activation of TRPV1 and TRPM3 ion channels is affected differently by the cholesterol content surrounding them in the plasma membrane.

We showed that MCD and SMase treatment inhibited the capsaicin-induced TRPV1 ion channel activation. These data are in accordance with our previous results. We have already described that TRPV1, TRPA1 and TRPM8 ion channel activation was decreased after lipid raft disruption both in TRG neurons and TRPV1- or TRPA1-transfected CHO cells by cholesterol depletion with MCD, different MCD derivatives and our own carboxamido steroid compound, by SM depletion with SMase or by the sphingolipid biosynthesis inhibitor myriocin. In TRG neurons, the lipid raft disruption with these compounds inhibited the capsaicin (TRPV1), AITC or formaldehyde (TRPA1) and icilin (TRPM8)-induced Ca^2+^-influx (Szoke et al., 2010a; Sághy et al., 2015c; 2018). Furthermore we proved the *in vivo* analgesic effect of MCD and SMase pretreatment in TRPV1, TRPA1 and TRPM8 ion channel-involving mouse models of pain and inflammation (Horváth et al., 2020a; Horváth et al., 2021a; Horváth et al., 2024a). However, MCD increased menthol-induced activation of TRPM8 on mouse dorsal root ganglion neurons and transfected cell line (Morenilla-Palao et al., 2009) and no effect of MCD was demonstrated on resiniferatoxin binding to rat C6 glioma cell membranes ((Bari et al., 2005). The present results are the first attempt to obtain evidence for the role of cholesterol content in nociceptive nerve terminals by measuring the Ca^2+^-dependent release of the sensory neuropeptide CGRP in response to the TRPV1 agonist capsaicin. Lower concentrations of MCD diminished ion channel activation on the terminals than on sensory neuronal cell bodies. We have already described similar observation after SMase treatment (Sághy et al., 2015a).

Disruption of lipid rafts by depleting cholesterol with MCD or cleaving, +a SMs by SMase did not cause, −d inhibition in CIM0216-evoked TRPM3 ion channel activation in TRG neurons. It is in accordance with our previous experiments, we have already described, that lipid raft decomposition by MCD, SMase and myriocin had no effect on PS-induced TRPM3 activation on native TRG neurons. Only our carboxamido-steroid compound was able to reach a small diminution in the PS-evoked TRPM3 activation on TRG, +R neurons. This compound was able to deplete cholesterol from the plasma membrane and reach the same effect in 10 µM concentration as MCD in 1000-times higher concentration, −r (Sághy et al., 2018). The unique characteristics of the TRPM3 ion channel can explain these findings. Like other TRP channels, TRPM3 is a cation-permeable ion channel located in the plasma membrane, but it has several distinct features, therefore, +e it differs from other TRP channel members (Wagner et al., 2008; Vriens et al., 2011b). Notably, TRPM3 contains a specific steroid-binding site, allowing activation by neurosteroids derived from cholesterol, such as PS, epipregnenolone sulfate, and dihydro-D-erythro-sphingosine (Grimm et al., 2005; Majeed et al., 2010; Drews et al., 2014). Previous studies have reported controversial results, MCD enhanced TRPM3 channel activation by PS in the contractile and proliferative phenotypes of mouse vascular smooth muscle cells, while decreased TRPM3 activation have been published after MCD/cholesterol complex administration in both human and mouse cells (Grimm et al., 2005; Drews et al., 2014). It has been described that TRPM3 formed a quaternary complex with an unknown auxiliary protein, making the channel resistant to lipid raft disruption by MCD, in this form the structure of the channel is similar to that of voltage-gated ion channels (Drews et al., 2014). It has also been described that CIM0216-evoked TRPM3 activation opens two distinct cation-permeable pores and induces the release of CGRP from sensory nerve terminals and of insulin from pancreatic islets (Held et al., 2015; Philippaert and Vennekens, 2015). Intrathecal injection of CIM0216 induced heat hypersensitivity in wild-type animals, but not in TRPM3 knock-out (KO) animals (Su et al., 2021). It has been shown that besides the central pore region it contains an alternative inwardly rectifying permeation pathway that can be activated by specific ligand combinations, such as by co-stimulation of PS and the antifungal drug clotrimazole or by stimulation of CIM0216 (Held et al., 2018) and the current of which can be distinguished from the canonical TRPM3 current (Vriens et al., 2011a; 2014). Evidence showed the involvement of the voltage-sensing domain of TRPM3 in the formation of this alternative ion permeation pathway (Held et al., 2018).

Therefore, we started to investigate whether MCD and SMase treatments caused inhibition in the function of voltage-gated ion channels in sensory neurons. MCD and SMase treatment did not influence the activation of the voltage-gated L-type Ca^2+^ channel activator FPL 64126 and veratridine on TRG neurons. Veratridine increases nerve excitability and due to the persistent Na^+^ current monophasic intracellular Na^+^ concentration and biphasic intracellular Ca^2+^ increase was shown in CA1 cells in acute pyramidal slices. The Ca^2+^ response was tetrodotoxin- and extracellular Ca^2+^-dependent, but ionotropic glutamate receptor-independent (Fekete et al., 2009). Investigating the tetrodotoxin-sensitive veratridine-induced Ca^2+^ responses in cultured mouse dorsal root ganglia neurons Mohammed et al. identified and characterized four different profiles representing subgroups of sensory neurons. These veratridine response profiles correlated with responses to the algesic markers capsaicin, AITC and α, β-methylene ATP (Mohammed et al., 2017).The effects of FPL 64126 are prolonged channel opening of single channels during depolarization and slowing of channel closure upon repolarization (Rampe et al., 1991; Zheng et al., 1991; Kunze and Rampe, 1992; Lauven et al., 1999; Fan et al., 2000). As FPL acts specifically through voltage-gated L-type Ca^2+^ channels without altering currents of other calcium channel types, it is widely used as a tool for investigating physiological or pathophysiological conditions affecting L-type channels, as well as for specific identification of L-type currents among different channel subtypes in neurons (Hardingham et al., 1997; Jinnah et al., 1999; 2003).

MCD treatment did not inhibit the electric field stimulation-induced CGRP release on sensory nerve terminals. We have already investigated earlier the effect of SMase on voltage-dependent Ca^2+^-influx after KCl administration on sensory neurons and sensory nerve terminals (Sághy et al., 2015b). The SMase treatment did not affect the percentage of responsive neurons to KCl nor the extent of the response. Few and contradictory results have come to light in this area. Disruption of lipid rafts increased the Ca_v_2.1 currents on cerebellar neurons (Davies et al., 2006), but MCD treatment decreased the activation kinetics of delayed K_v_3.1 currents on a neuronal cell line (NG108-15) (Huang et al., 2011). The KCl-evoked Ca^2+^-influx involves several K^+^ and Ca^2+^ channels in and as we previously reported, the overall response remained unaltered (Szoke et al., 2010b).

The potential role of cholesterol-TRP interactions and the cholesterol level in the plasma membrane in TRP ion channel activation were in the focus of other research group also (Startek et al., 2019; Startek and Talavera, 2020). The significance of the lipids in conformation changes of TRP ion channels between active, inactive or desensitized states were supported by CryoEM investigations (Zhang et al., 2021). These data confirmed our hypothesis that channel activation and inhibition have been influenced by the direct protein-lipid hydrophobic interactions. Change the position of TRP ion channel in the plasma membrane is making the binding sites unavailable for agonist compounds (Horváth et al., 2022). In the present study, we compared the activation mechanisms of TRPV1 and the TRPM3 ion channel, which is similar to the structure of voltage-gated ion channels.

All-in-all, the above atomic level calculations revealed that the loss of ion conductance (see Section 3.1) after the depletion (removal) of cholesterol from the membrane of TRPV1 can be explained by the higher accessibility of more loosely bound cholesterol molecules, that are easier to remove by complexation with cyclodextrin molecules. The removal of cholesterol from the membrane of TRPM3 and Ca_v_ 1.3 did not result in loss of ion conductance, as a large cluster of strongly bound cholesterol molecules are buried in the transmembrane helices that are harder to remove by complexation. The case of Ca_v_ 1.3 is in between TPRV1 and TRPM3, as a large cluster of loosely bound cholesterol molecules are found in the transmembrane region of the ion channel, hidden from the extracellular surface.

It is concluded that the lipid membrane environment modulates the function of TRPV1 channels, but not TRPM3 channels and voltage-gated L-type Ca^2+^ channels in primary sensory neurons. The hydrophobic interactions between lipid raft and TRP ion channels have significant importance in channel opening properties. Therefore, this approach might open novel perspectives in pain management most importantly at the periphery.

## Data Availability

The raw data supporting the conclusions of this article will be made available by the authors, without undue reservation.
